# Beta-band desynchronization in the human hippocampus during movement preparation in a delayed reach task

**DOI:** 10.1007/s00221-025-07124-6

**Published:** 2025-06-23

**Authors:** Xiecheng Shao, Ryan S. Chung, Shivani Sundaram, Roberto Martin Del Campo-Vera, Jonathon Cavaleri, Selena Zhang, Adith Swarup, Alexandra Kammen, Miguel Parra, Xenos Mason, Christi Heck, Charles Y. Liu, Spencer S. Kellis, Brian Lee

**Affiliations:** 1 Department of Neurological Surgery, Keck School of Medicine of USC, University of Southern California, 1200 N State Street, Suite 3300, Los Angeles, CA 90033 USA; 2 USC Neurorestoration Center, Keck School of Medicine of USC, Los Angeles, CA USA; 3 Department of Neurology, Keck School of Medicine of USC, University of Southern California, Los Angeles, CA USA; 4 Keck School of Medicine of USC, University of Southern California, Los Angeles, CA USA; 5 Department of Biomedical Engineering, Viterbi School of Engineering of USC, University of Southern California, Los Angeles, CA USA

**Keywords:** Beta-band power, Stereotactic electroencephalography (SEEG), Neural modulation, Hippocampus, Delayed reach task, Movement planning

## Abstract

Though the hippocampus is known for its role in memory and navigation, growing evidence has suggested that it is involved in voluntary movement. By investigating whether the hippocampus is active during movement preparation, we can understand its role in motor control. In this study, we utilized a Delayed Reach task to measure beta-band (13–30 Hz) amplitude changes in the human hippocampus during movement preparation. We hypothesized to observe beta-band event-related desynchronization (ERD) during the movement preparation phases. Eleven patients diagnosed with drug-resistant epilepsy were implanted with stereoelectroencephalographic (SEEG) depth electrodes and SEEG signals were recorded. We pre-processed the raw neural signals using the zap-line noise removal technique and re-referenced the data using a novel weighted electrode shaft re-referencing technique. The beta-band Power Spectral Density (PSD) was calculated using multi-taper spectral analysis and trial averaged PSD between task phases was compared using a cluster-based permutation test. We then compared modulation between ipsilateral vs. contralateral contacts using the group-level Yate’s z-test. 91% of participants and 46.8% of hippocampal gray matter contacts (n = 149) exhibited significant beta-band ERD during the Delay phase compared to baseline. During the Response phase, 100% of participants and 69.8% of hippocampal gray matter contacts exhibited significant beta-band decreases. We observed no significant difference between ipsilateral and contralateral contacts (*p* > 0.05). This study is the first to demonstrate hippocampal beta-band modulation during movement preparation, implying that the hippocampus may be involved during the movement processing.

**Supplementary Information:**

The online version contains supplementary material available at 10.1007/s00221-025-07124-6.

## Introduction

Even the simplest movement consists of many different phases and stages of brain involvement before its final execution. The brain structures involved in motor control have been widely investigated, and most studies have implicated the primary motor cortex, pre-motor areas, and cerebellum as the primary structures that control movement (Graziano et al. 2002; Matsuzaka et al. 1992; Popa et al. 2016; Schieber and Hibbard 1993). One common phase studied in voluntary movement is movement preparation, which is the period of time before motor initiation in which a subject plans their movement. Yeom et al. (2020) used magnetoencephalography (MEG) to demonstrate increased connectivity between motor-related regions during and before movement execution, suggesting that the neural processes required for motor control initiate prior to the actual onset of movement (Yeom et al. 2020). In addition, they showed that the brain networks involved in movement planning are separate from those involved in motor execution (Yeom et al. 2020). Given that this study found network involvement of regions not traditionally considered as motor areas, it is important to further understand and investigate the extent to which these structures are involved in movement planning.

A growing body of research has demonstrated hippocampal activity during movement. In addition to its known roles in declarative memory (Rolls 1996; Scoville and Milner 1957; Squire et al. 2004) and spatial navigation (Buzsáki and Moser 2013; Ekstrom et al. 2005), the hippocampus is implicated in learning motor sequences (Albouy et al. 2008; Burman 2019; Fernández-Seara et al. 2009; Gheysen et al. 2010), responding to movement perturbations (Kerr et al. 2017), and interacting with motor-related areas such as the motor cortex and cingulate cortex during a motor learning task (Shah et al. 2019). In one study using rats, researchers found that theta-band activity in the hippocampus, which is known to be involved in motor programming (Bland 1986; Morris and Black 1978; Vanderwolf 1969), was responsible for initiating and guiding movement in response to sensory stimuli (Oddie 1998). Our previous study also found a decrease in beta-band power, a phenomenon commonly associated with motor control (Buzsáki and Draguhn 2004), during movement execution in the hippocampus during a direct reach task (Del Campo-Vera et al. 2020). These findings collectively suggest that the hippocampus is involved in movement, prompting the question of whether the hippocampus also contributes to motor preparation. One common modality that is primarily used to study motor preparation is the Delayed Reach task, in which participants identify a reaching target but must withhold movement until a “Go” signal appears (Ames et al. 2019). Most studies utilizing Delayed Reach tasks focused on motor anticipation in the motor cortex and premotor cortex (Alexander and Crutcher 1990; Crammond and Kalaska 1994; Mauritz and Wise 1986; Tanji and Evarts 1976; Wise and Mauritz 1985). Furthermore, several studies employing Delayed Reach tasks have shown that movement is generated from a preparatory state, characterized by distinct neural signaling that triggers the onset of that movement (Churchland et al. 2010; Michaels et al. 2016; Shenoy et al. 2011; Sussillo et al. 2015).

Various imaging and recording modalities have been used to observe how brain signals evolve during movement. Previous studies have used intracortical multielectrode arrays (Aflalo et al. 2015; Afshar et al. 2011; Chapin 2004; Churchland et al. 2007; Kellis et al. 2010), functional magnetic resonance imaging (fMRI) (Burianová et al. 2020; Crotti et al. 2022; Klein et al. 2022; Ma et al. 2023; Prokopiou et al. 2022), MEG (Yeom et al. 2020), electroencephalography (EEG) (Bennet and Reiner 2022; Calabrò et al. 2017; Dieckgraeff et al. 2011; Pinet et al. 2016), and electrocorticography (ECoG) (Choi et al. 2018; Jung et al. 2021; Leuthardt et al. 2004) to investigate motor planning and execution across different brain regions. There has been a shift to using stereoelectroencephalography (SEEG) to record neural signals for seizure monitoring in clinical settings, particularly in epilepsy surgery, and subsequently in research studies, such as those involving movement tasks (Yamamoto 2020). SEEG offers several advantages, including relatively high temporal resolution and a low signal-to-noise ratio (Li et al. 2018; Youngerman et al. 2019), and allows intracranial recordings with fewer infectious and hemorrhagic complications compared to other invasive modalities, such as subdural grid placement (Mullin et al. 2016). With modern surgical implantation techniques, accurate placement of SEEG leads can yield valuable insights when studying hippocampal electrophysiology (Del Campo-Vera et al. 2020; Tang et al. 2021).

In the brain, oscillations within different frequency bands and structures have been attributed to specific processes (Cantero and Atienza 2005; Knyazev 2007; Nunez 2000). For example, beta-band oscillations (13–30 Hz) (Buzsáki and Draguhn 2004) are modulated during cognitive (Jensen and Bonnefond 2013; Sheth et al. 2009; Wagner et al. 2016), auditory (Cirelli et al. 2014; Fujioka et al. 2015; Todorovic et al. 2015), and motor activity. Various studies have demonstrated decreases in beta-band power, also referred to as beta-band event-related desynchronization (ERD), during movement execution in the motor cortex (Baker et al. 1997; Kilner et al. 1999; Sanes and Donoghue 1993). Other studies have also found beta-band ERD during movement planning and execution in the motor cortex (Klostermann et al. 2007; Tzagarakis et al. 2010), basal ganglia (Alegre et al. 2005; Courtemanche et al. 2003), thalamus (Paradiso et al. 2004), cerebellar nuclei (Aumann and Fetz 2004), posterior parietal cortex (Brovelli et al. 2004; MacKay and Mendonça, 1995), and contralateral peri-Rolandic region (Formaggio et al. 2008; Murthy and Fetz 1996; Pfurtscheller and Berghold 1989; Pfurtscheller and Neuper 1997; Sanes and Donoghue 1993; Schnitzler et al. 1997). To investigate if there is a causal relationship between beta-band power and movement speed, Khanna and Carmena (2017) trained rhesus macaque monkeys to achieve a predetermined level of beta-band power in the motor cortex prior to performing arm reaches (Khanna and Carmena 2017). They found that the degree of beta-band power reduction during movement execution correlated with the onset speed of movement, and that a reduction in baseline beta-band power before movement significantly increased movement onset time (Khanna and Carmena 2017). Similarly, Kilner et al. (1999) examined beta-band power during a Delayed Reach task and found beta-band ERD in the motor cortex during the Response phase, suggesting that the beta-band inhibits movement (Kilner et al. 1999). Our lab also used a Delayed Reach task and found neural tuning during the delay (planning) period in structures like the inferior parietal cortex and supramarginal gyrus (Gilbert et al. 2022).

In this study, we aim to characterize the dynamics of beta-band power in the hippocampus during movement preparation by using a Delayed Reach task and a novel re-referencing approach to address uneven amplitudes across multiple contacts in a single electrode. We focused on the beta-band given its role in voluntary movement throughout many other brain structures, and selected the Delayed Reach task for its ability to experimentally isolate motor planning from execution (Ames et al. 2019). In line with previous findings, we hypothesize that there will be beta-band ERD in the hippocampus during movement execution in the Response phase and during motor preparation in the Delay phase of a Delayed Reach Center-Out task.

## Experimental procedures

### Participants

**Fig. 1:**
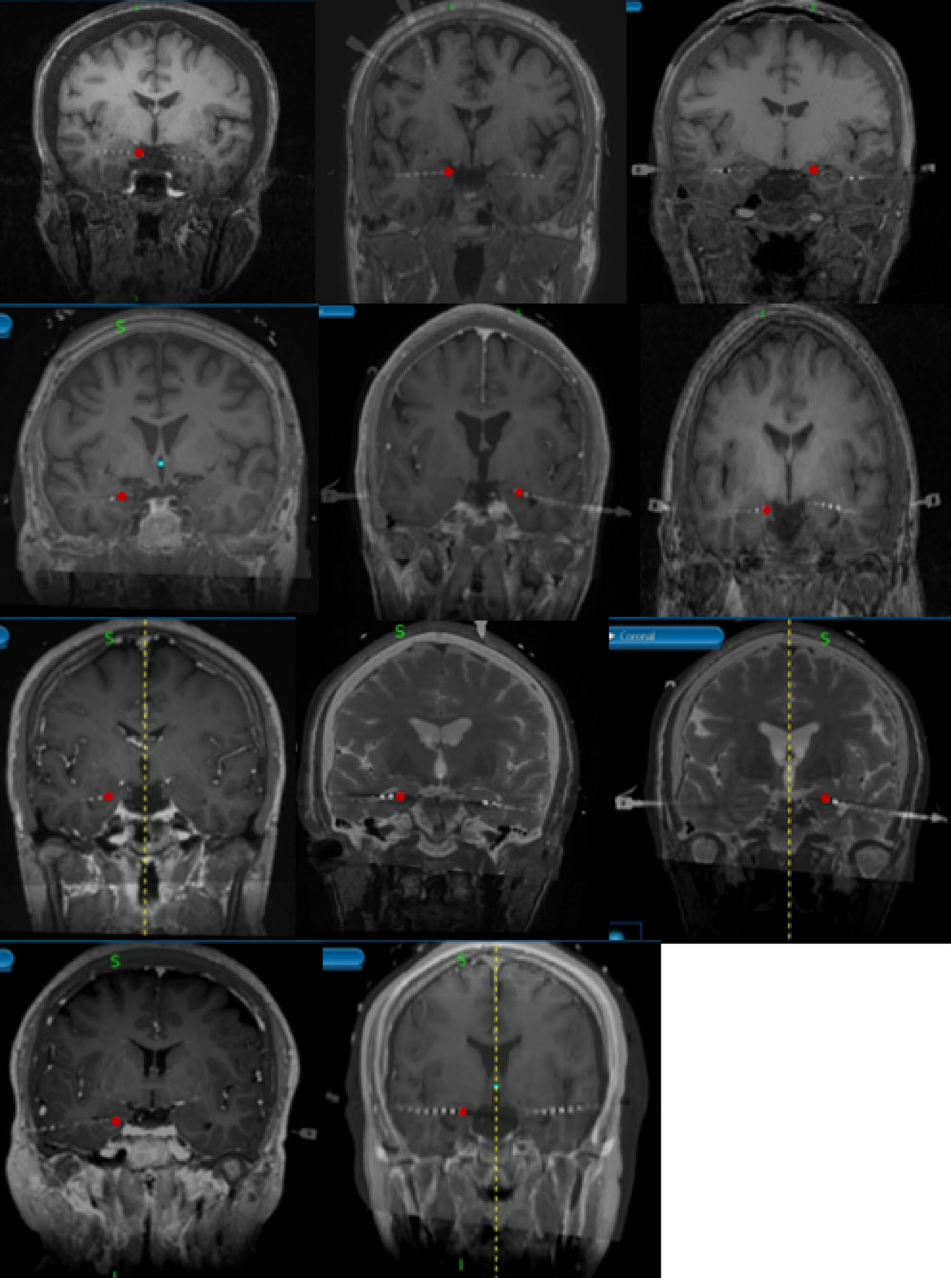
CT and MRI merged images using Medtronic StealthStation of 11 patients. The red dot in each figure represents the most distal hippocampus contact in the electrode

Eleven participants (5 males, 6 females) aged 21 to 51 years (mean 35.8 years), were implanted with SEEG depth electrodes (Ad-Tech Medical Instrumentation Corporation, Oak Creek, WI, USA) for seizure localization. All patients were diagnosed with drug resistant epilepsy, were admitted for seizure localization and underwent surgery to position SEEG leads in various regions of the brain, including the hippocampus, according to clinical criteria. The locations of seizure onset are indicated in Table 1. All enrolled participants provided informed consent for their participation in this study (Study ID: HS-17-00544), which was approved by the Institutional Review Board (IRB) of the University of Southern California (USC) Health Science Campus. The number of electrodes and implant locations were determined clinically for each patient prior to recruitment into the study based upon magnetic resonance imaging, positron emission tomography scans, video-EEG monitoring, and seizure semiology. Each case underwent thorough discussion with epileptologists, neuroradiologists, and neurosurgeons at the USC Comprehensive Epilepsy Center. Ten of the eleven participants had depth electrodes implanted bilaterally in the hippocampus, and the remaining participant had depth electrodes implanted unilaterally in the left hemisphere (contralateral to dominant hand). Detailed information about electrode placement for each participant, including laterality and number of leads implanted, is summarized in Table 1. Merged MRI and CT images of hippocampal electrode contact placement are included in Fig. 1.

**Table 1:** Demographics of 11 participants

Participant	Handiness	Seizure onset zone	Gender	Age
1	R	Right insula and frontal Operculum	F	45
2	R	Right inferior frontal	F	40
3	N.A	R anterior hippocampus	M	37
4	R	R mesial temporal	F	21
5	R	R mesial temporal	M	26
6	R	Right hippocampal head	F	21
7	R	Right basal temporal	F	43
8	R	Right hippocampal tail	M	32
9	R	Left mesio temporal	M	39
10	R	L posterior orbitofrontal	M	39
11	R	L temporal	F	51

Most of the participants use right-hand dominant, and the task was performed by the right hand in all participants. 6 out of 11 participants are female. 3 out of 11 are between 21 and 30 years old, 4 out of 11 are between 31 and 40, 3 out of 11 are between 41 and 50, and 1 out of 11 is between 51 and 60. Three of the participants have seizure onset zones in the hippocampal region

### Electrodes and recording equipment

The recording equipment used in this study consisted of two types of electrodes and a neural signal processor. The first type of electrode was the Spencer probe depth electrode, featuring platinum contacts with a length of 2.29 mm and came in two variants: part numbers RD08R-SP05X-000 with 8 contacts spaced 4 mm apart, and RD10R-SP05X-000 with 10 contacts spaced 5 mm apart. The second type was the macro–micro depth electrode, which included 16 contacts with 6 macro and 10 micro contacts, platinum contacts with a 1.3 mm diameter and a length of 1.57 mm (part number MM16C-SP05X-000), and a spacing of 5 mm. Recordings from only the macro-type contacts were used in this study. All electrodes were obtained from Ad-Tech Medical Instrumentation Corporation, Oak Creek, WI, USA. Neural electrical activity was amplified, digitized, and recorded using the NeuroPort Neural Signal Processor (Blackrock Microsystems, Salt Lake City, UT) at 2000 samples per second with 16 bits per sample (250 nV resolution) and filtered with 1st-order high-pass (0.3 Hz) and 3rd-order low-pass (7500 Hz) analog Butterworth filters. A 4th order Butterworth anti-aliasing filter was also applied to the raw data with a cut-off frequency of 500 Hz.

### Delayed reach center-out task

**Fig. 2:**
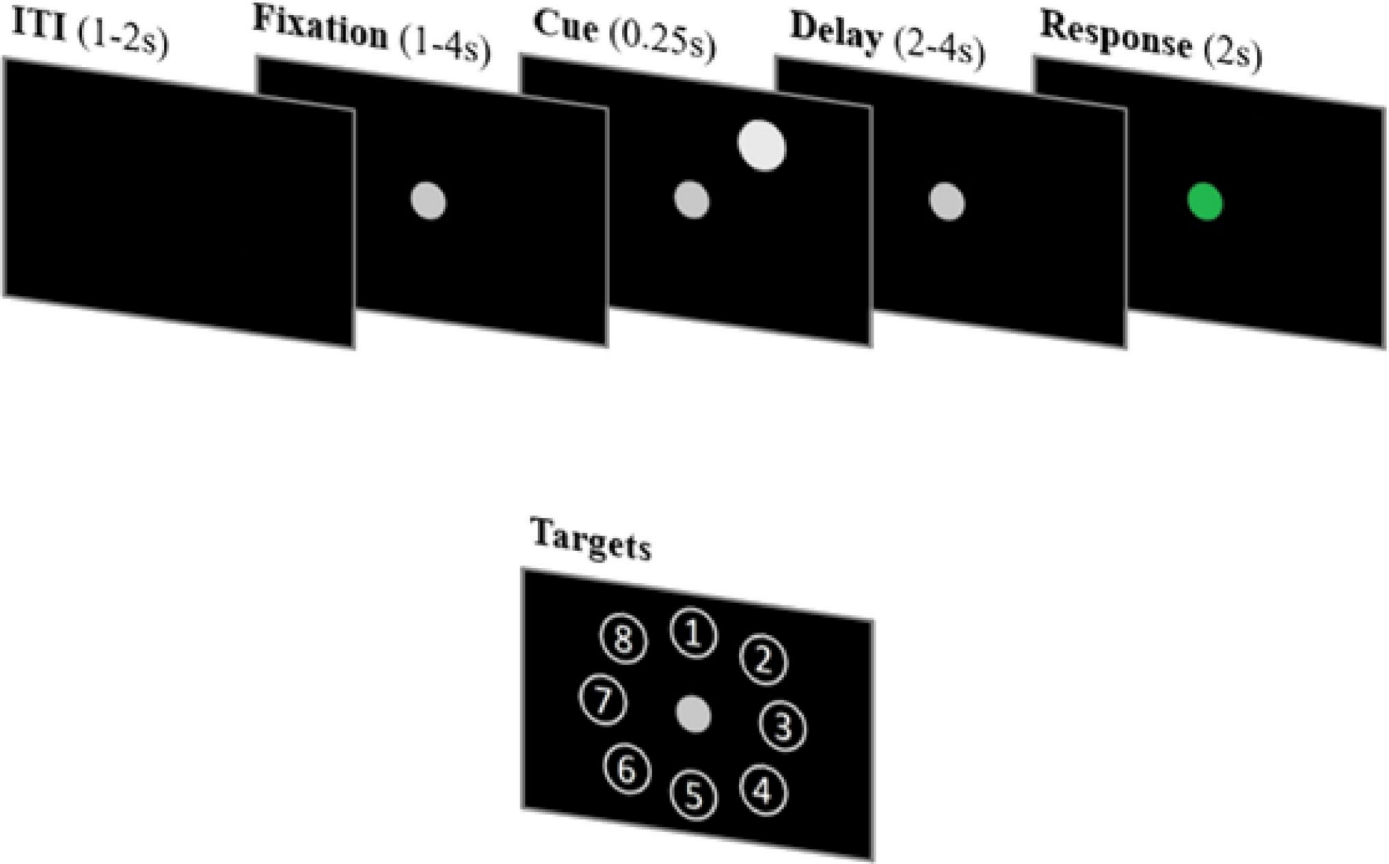
Delayed Reach task diagram. The participant was instructed to fix their hand at the dot during the Fixation phase, Cue phase, and Delay phase, and they were told to move during Response phase. During Cue phase, one dot appeared at a location outside of the center dot in 1 of 8 different directions, which is shown at the bottom of the figure. This dot disappeared at the start of the Delay phase. During the Response phase, the participant was instructed to double tap at the cue dot location

The Delayed Reach Center-Out task was divided into five phases: Inter-Trial Interval (ITI) (1–2 s), Fixation (1–4 s), Cue (0.25 s), Delay (2–4 s), and Response (max 2 s). During the ITI phase, participants were instructed to position their right hand at the center of the screen. During the Fixation phase, participants pointed at a gray dot (9.53 mm radius) located at the center of the screen. During the Cue phase, a white circle (15.88 mm radius) was shown at one of eight locations equally spaced around the fixation point (114.3 mm center to center), while the participants continued to maintain their gaze and hand on the fixation dot. During the Delay phase, the cue disappeared, and participants continued to keep their hand still. Finally, in the Response phase, participants moved their hand towards the target shown during the Cue phase. The task consisted of a total of 64 trials, and hand trajectories were recorded using an accelerometer attached to the back of the gloves that participants wore on their performing hand. The task was programmed using MATLAB (2018b, The MathWorks, Inc., Natick, Massachusetts, USA) with the Psychophysics Toolbox Version 3 (PTB-3) and presented on a touch-screen monitor (21.5-inch LED-backlit, 1920 × 1080 pixels, 250 cd m − 2 luminance, S2240Tb, Dell Inc., Round Rock, TX, USA). The Delayed Reach experimental task is illustrated in Fig. 2. A pillow was placed under all participants' forearms to provide a close-to-rest position while they maintained the hand position, thereby minimizing muscle fatigue and potential motion artifacts that could arise from sustaining an unnatural posture during the extended delay periods.

### Behavioral data collection

We calculated several behavioral performance metrics, including average delay time, average trial time, average response time, total trials, and success rate. Successful trials were defined as those trials where the participant both correctly double-tapped the target region and did so efficiently (response time < 5 s). Next, we calculated the average time only using the defined successful trials. The average trial time was measured from the start of the ITI to the end of the Response phase. The average response time was defined as the duration from the response onset to the completion of the hand movement. The success rate was calculated as the percentage of trials correctly executed within the designated response window.

### Data analysis

#### Line noise removal

The ZapLine noise removal technique (python package: meegkit) was employed to eliminate noise at 60 Hz and its harmonics (De Cheveigné, 2020). This technique was chosen because it does not create artifacts during large transitions in the amplitude during short time windows, like a spike, as occurs when using a notch filter (Kirac et al. 2016). Additionally, it is able to address fluctuations in amplitude and phase within line noise components, a potential concern with sinusoidal line fitting (De Cheveigné, 2020).

#### Re-referencing method

Since the contacts in the same SEEG electrode shaft can be in both gray matter and white matter, we observed substantial variations in signal amplitudes across different contacts in the same electrode shaft. The task of removing common noise across many contacts implanted at distant locations presented a challenge. Using standard Electrode Shaft Referencing (ESR) methods would risk contaminating the low amplitude signals, which are usually recorded in the contacts within white matter with high amplitude signals. Therefore, to cancel out the common noise while preventing high amplitude contacts from contaminating low amplitude contacts, a novel weighted electrode shaft re-referencing method was developed. In a normal ESR method, the common noise is calculated as the average of all the contacts in the same electrode shaft. Therefore, a contact that has a higher amplitude will tend to contribute more to the common noise signal. When the common noise signal was subtracted from the original signal, the contact with the lower amplitude will have a comparatively larger effect. To solve this, the Root Mean Square was used, a representation of the amplitude of activity on a given contact, to scale the weight of each channel when calculating the common noise. Specifically, larger RMS values should lead to lower weights. To quantify this intent, ${\widehat{RMS}}_{i}$ was defined as follows:

$$ \widehat{RMS}_{i} = - 1*RMS_{i} + 2*{\text{max}}\left( {RMS_{ele} } \right), $$

where $RM{S}_{i}$ was the RMS value computed for the *i*th contact, and $RM{S}_{ele}$ is the vector of RMS values for all contacts in the same electrode shaft as *i*. The quantity $2*\text{max}(RMS)$ was included to ensure positive ${\widehat{RMS}}_{i}$. The weight ${W}_{i}$ applied to the *i*th channel’s neural activity during software re-referencing was then defined in terms of ${\widehat{RMS}}_{i}$ and $RM{S}_{j}$, the RMS value computed for the *j*^th^ contact across all contacts in the same shaft:

$$ {\text{Weight}}:\,W_{i} = \left( {\frac{1}{{\mathop \sum \nolimits_{j = 0}^{NContacts} \widehat{{RMS_{j} }}}}} \right)*\widehat{{RMS_{i} }} $$

For each phase in each trial, the signals in the same electrode shaft were re-referenced using the following equation where *C* is the total number of contacts in an SEEG electrode:

$$ \hat{s} = s{ } - \mathop \sum \limits_{i = 0}^{C} W_{i} s_{i} $$

A common characteristic of epilepsy is the interictal spike, which was observed to some degree in all patients. Only channels with no interictal spikes were included when calculating the weights. We identified interictal spikes using a fixed thresholding method (high pass filter at 30 Hz, followed by a threshold of 30 μV). A detailed comparison between normal ESR and weighted ESR is provided in the supplementary material.

#### Trial selection

Signal quality was assessed under the guidance of our epileptologists. Detailed manual reviews were conducted to identify and exclude trials contaminated with interictal spikes. Furthermore, trials with a Response phase duration less than 0.5 s or longer than 3 s were removed, as too short trials indicate that the participant moved before the start of the Response phase and too long trials indicate that the participant was not paying attention during tasks. If the total number of successful trials in a run of 64 trials was less than 50% of the total after the removal of the bad trials, the entire run was discarded.

A duration of 0.5 s at the start of both the Fixation and Delay phases was selected for analysis. This time window was selected at the center of these phases to ensure accurate data capture for analysis. The Cue phase, which had a duration of only 0.25 s, was selected in its entirety for comparison. However, due to its shorter duration, the results from the Cue phase are not discussed in detail in the subsequent sections. The analysis window for the Response phase was also set at 0.5 s, but the selection was made at the end of the phase (the end of the phase marked the time at which the participant touched the target) to ensure movement was captured within the analysis window. The ITI phase was set at a duration of 0.5 s, starting 0.8 s into the phase to ensure that no movement occurred within the analysis window.

#### Multitaper power spectral density analysis

The multitaper spectral analysis method (using python package multitaper (Prieto 2022)) was selected due to its anti-leakage properties. For the PSD analysis, we examined 0.5 s segments from each task phase, using 5 tapers and a time-bandwidth product of 3. The implementation in the multitaper package sets the nfft parameter to use zero-padding to discretize the frequency axis in approximately 0.3 Hz. The mean PSD for each channel was calculated within the beta-band, and the trial average was determined after excluding outlier trials, whose criteria will be discussed in the next paragraph. The 95% confidence interval was computed using bootstrap resampling (N = 10,000).

Outlier trials for each channel were excluded from comparison. Two types of outliers were identified: time series outliers and frequency series outliers. For time series outliers, the RMS was calculated for each trial, and trials with an RMS exceeding 1.5 times the interquartile range of RMS of all trials were excluded. Each trial was also examined for interictal spikes using the spike detection method described earlier, and trials exhibiting interictal spikes were removed. For frequency series outliers, the PSD calculated for the beta-band was used. Trials with a PSD exceeding 1.5 times the interquartile range were also removed.

Additionally, spectrograms were calculated across the frequency range of 1–100 Hz using the same multitaper spectral analysis method (python package multitaper (Prieto 2022)) with 5 tapers and a time-bandwidth product of 3, applied to a 90% overlapping 0.3 s sliding window. Zero-padding was automatically employed by the package resulting in a discretization of the frequency axis to 0.3 Hz each time step. Power within each frequency bin was z-score normalized relative to the entire trial period for visualization purposes.

#### Tests of statistical significance

Because neural modulation may occur only within a small frequency bin within the beta-band frequency range (Chen et al. 2020; del Campo-Vera et al. 2022), comparing each frequency bin requires addressing the problem of multiple comparisons. However, using any p-value correction method with many comparisons will decrease the sensitivity of the test. Therefore, in order to test the significant difference in beta-band power between two phases (P_Fixation_ > P_Delay_ and P_Fixation_ > P_Response_) within the same contact, the cluster-based permutation test (N = 5000) was chosen because it not only addresses the multiple comparisons problem but also considers the spectral information (Maris and Oostenveld 2007).

The test statistics for each comparison were obtained as follows (Maris and Oostenveld 2007): (1) for each frequency bin, a Wilcoxon signed-rank test was performed between the same frequency bin in different phases and the z-score for the test statistic was obtained; (2) A z-score threshold of 1.96 for two-tailed tests (when comparing P_Delay_ vs. P_Response_ H1: P_Delay_ ≠ P_Response_) or 1.645 for one-tailed tests (when testing H1: P_Fixation_ > P_Delay_ or P_Fixation_ > P_Response_) was applied, and consecutive frequency bins that have z-scores exceeding this threshold were identified as a cluster; and 3) The summation of each cluster's z-score was calculated and the maximum of the summation across all clusters was used as the cluster-level test statistic.

The permutations were computed across trials within each subject. Specifically, for each of the 5000 permutation iterations, we randomly swapped the condition labels (e.g., Fixation vs. Delay) for each trial and recalculated the test statistics following the three steps described above. The p-value was calculated by examining the proportion of cluster-level test statistics after permutation that is smaller than the cluster-level test statistics using the original dataset. Frequency range of significance is determined by the largest cluster if significant difference is observed between two phases.

Given our small cohort (11 participants), which is underpowered for group-level statistical tests (Cohen’s f = 0.25, 80% power, alpha = 0.05, G Power 3.1 requiring 28 subjects for a mixed effects ANOVA), we analyzed individual subject data to identify modulation patterns. For individual patient analysis, only contacts demonstrating significant power differences (*p* < 0.05) between phases were considered indicative of significant modulation, and the largest cluster was reported with min–max frequency range.

To compare differences in electrode locations during beta-band modulation, a group level Yates-corrected z-test (Hoffman 2019) was used between ipsilateral and contralateral electrodes in gray matter (n_ipsi = 64, n_contra = 85).

## Results

### Behavioral performance

Behavioral data (Table 2) revealed consistent performance in terms of success rate and movement time across participants. The average trial time was 8.80 ± 1.15 s, with a range of 6.47 to 10.18 s. The average delay time was 2.53 ± 0.63 s, ranging from 1.52 to 3.17 s. The average response time was 1.47 ± 0.21 s, ranging from 1.22 to 1.98 s. Overall, participants achieved a high success rate of 87.85 ± 11.59%, with individual success rates spanning from 55.93% to 98.39%. Total trials ranged from 59 to 124 trials with an average of 72.64 ± 24.23.

**Table 2:** This table presents detailed behavioral performance metrics for each participant, including performing hand for each session, success rate, mean delay time, mean movement time, mean trial time, the total number of trials and trials remained after outlier removal

Patient	Handiness	Success rate (%)	Mean delay time (s)	Mean movement time (s)	Mean trial time (s)	Total trials	Trials after outlier removal
1	Right	98.39	2.04	1.44	8.51	62	59
2	Right	95.16	2.09	1.47	8.65	62	56
3	Right	80.65	2.01	1.59	8.89	62	49
4	Right	88.52	1.68	1.38	6.75	61	52
5	Right	93.55	1.52	1.33	6.47	124	113
6	Right	96.77	2.94	1.22	9.23	124	116
7	Right	55.93	3.13	1.98	10.18	59	32
8	Right	87.1	3.1	1.67	9.87	62	52
9	Right	88.33	3.05	1.16	9.44	60	50
10	Right	98.39	3.17	1.46	9.83	62	59
11	Right	83.61	3.15	1.5	9.03	61	50
Avg		87.85	2.53	1.47	8.8	72.64	62.55
SD		11.59	0.63	0.21	1.15	24.23	25.45

All sessions right-handed were used, and the success rate represents the percentage of correctly executed trials. Mean delay time refers to the average duration between the end of cue and the initiation of movement, while mean movement time indicates the average duration of the movement phase. Mean trial time is the entire trial duration from start to finish. The total number of trials conducted for each participant is also listed. The table includes the average (AVG) and standard deviation (SD) for each metric across all participants

### Event related desynchronization during the delay phase in the beta-band

We observed a beta-band ERD in the hippocampus during the Delay phase, relative to the Fixation (baseline) phase, in at least one channel in 10 out of 11 participants (90.9%) using the Wilcoxon signed-rank test (p < 0.05) (Table 3). In addition, 70 out of the total 149 Gy matter contacts implanted across all participants (46.8%) showed this same modulation (Table 3).

**Table 3:** Statistical table of detailed comparison of beta-band modulation between the Delay and Fixation phases

Participant	Hand	Total	Loc	Hem	Contacts (%)	Avg freq	Max *p* value	Freq range
1	Right	7	Hippo	Left	85.71	21.76	0.045	13.0–30.0
	Right	8	Hippo	Right	87.50	21.50	0.001	13.0–30.0
2	Right	8	Hippo	Left	75.00	25.83	0.049	21.96–30.0
	Right	7	Hippo	Right	85.71	25.67	0.048	25.67–25.67
3	Right	4	Hippo	Left	75.00	28.30	0.042	22.89–30.0
	Right	4	Hippo	Right	75.00	27.27	0.049	23.81–30.0
4	Right	10	Hippo	Left	30.00	21.29	0.049	13.0–30.0
	Right	9	Hippo	Right	33.33	20.52	0.049	13.0–30.0
5	Right	9	Hippo	Left	33.33	27.06	0.046	26.6–27.52
6	Right	4	Hippo	Left	75.00	25.88	0.050	21.34–30.0
	Right	9	Hippo	Right	77.78	25.83	0.049	23.81–28.45
7	Right	11	Hippo	Left	9.09	29.38	0.050	28.76–30.0
	Right	5	Hippo	Right	0.00	N.a	N.a	Nan–nan
8	Right	2	Hippo	Left	0.00	N.a	N.a	Nan–nan
	Right	2	Hippo	Right	100.00	26.91	0.036	23.81–30.0
9	Right	6	Hippo	Left	0.00	N.a	N.a	Nan–nan
	Right	3	Hippo	Right	0.00	N.a	N.a	Nan–nan
10	Right	11	Hippo	Left	45.45	27.32	0.048	21.34–30.0
	Right	8	Hippo	Right	0.00	N.a	N.a	Nan–nan
11	Right	13	Hippo	Left	38.46	22.33	0.048	13.0–28.14
	Right	9	Hippo	Right	55.56	16.46	0.049	13.0–25.36
Avg		7.1			46.8	24.58		19.90–29.07

A cluster-based permutation test was used to calculate the percentage of contacts in each location with significant modulation. The average percentage of contacts that showed modulation is 46.76%, with an average frequency bin of 24.58 Hz. The frequency range was calculated using the mean of the minimal and maximal frequency range of each contact within that location

**Fig. 3:**
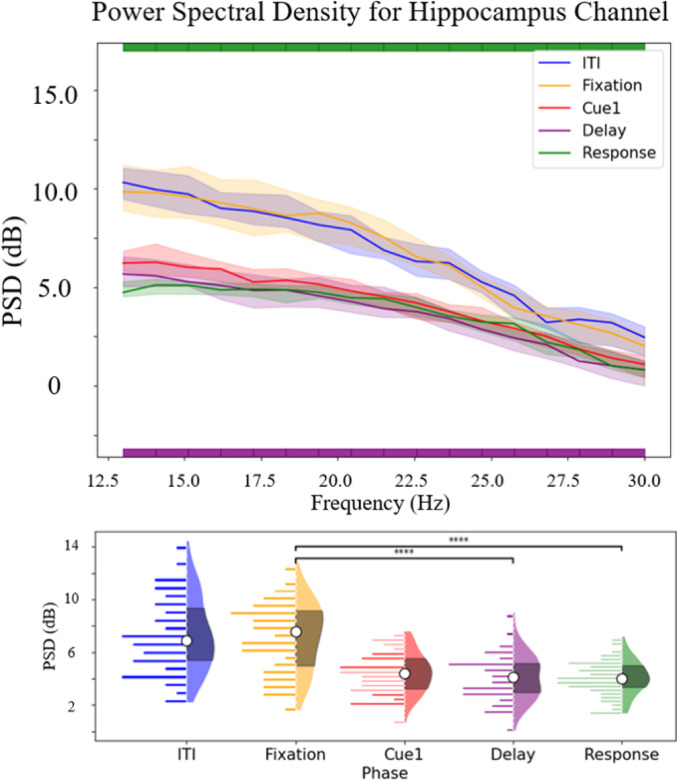
Representative illustration of beta-band power modulation during the Delay and Reponse phases as determined by PSD analysis of participant 1 in the most medial Left Anterior hippocampus (LAH) location. On the top pannel, PSD was calculated using multitaper spectral analysis. The confidence interval was calculated using a bootstrap (N = 10,000) resampling method. The sigficance test of each frequency bin (~ 0.3 Hz per bin) was calculated using a cluster-based permutation test. We observed a decrease in beta-band power in all frequency bins in this channel. The bottom panel shows a violin plot/histogram combination of the PSD of the median of beta power PSD of each trial during each phase. The cluster-based permutation test was used to test the difference between different phases. We observed a significant decrease in beta-band power between the Fixation and Delay phases (*p* < 0.001) and the Fixation and Response phases (*p* < 0.001)

Contiguous beta-band frequency bins with statistically significant power differences between the Delay and Fixation phases after adjusting for the false discovery rate are presented in Table 3. This includes the average frequency range of statistically significant trial-averaged power modulation in addition to the number of electrode contacts in hippocampal gray matter that showed statistically significant power modulation for each patient. On an individual level, Fig. 3 shows the PSDs computed during each phase for participant 1, exhibiting a representative decrease in beta-band power in the Delay phase.

**Fig. 4:**
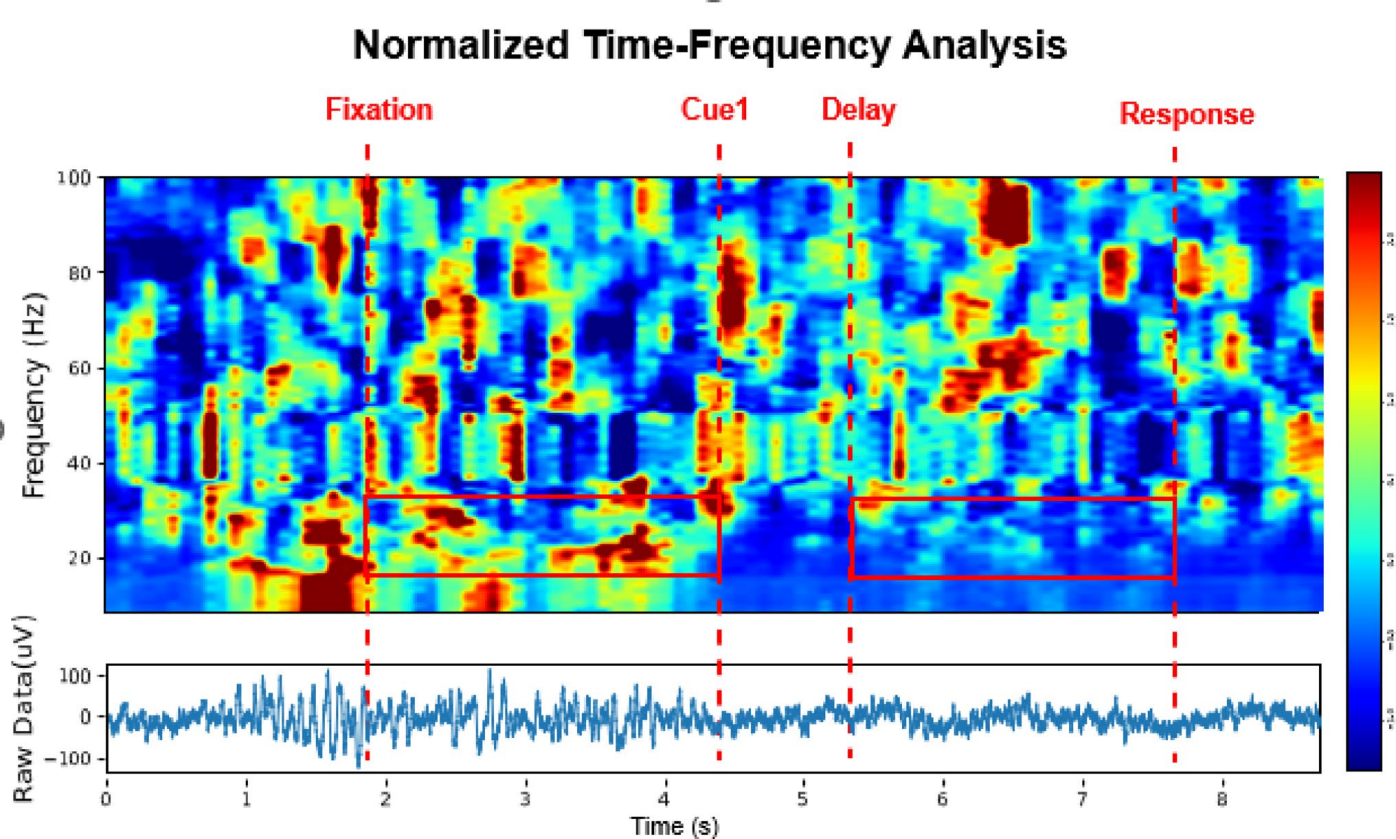
The upper panel is a time–frequency spectrogram recorded from a left hippocampus electrode in participant 1. The spectral power calculation was performed using multitaper analysis (3 tapers, time-bandwidth product NW = 5), with a 0.3s sliding window with 90% overlap. Power values were z-score normalized relative to the complete trial duration for each frequency bin, with the color scale showing standard deviations from the mean. Vertical red dashed lines mark the transitions between task phases (Fixation, Cue1, Delay, and Response). The beta frequency range (13–30 Hz) is highlighted by a red box during the Fixation and Delay phases. The lower panel displays the corresponding raw neural signal used for the time–frequency analysis above. Signal preprocessing included line noise removal at 60 Hz and its harmonics using the ZapLine noise removal, followed by bandpass filtering (0.3–200 Hz) with a 5th-order Butterworth filter

Analysis of time–frequency dynamics revealed changes in neural activity throughout different task phases (Fig. 4). The spectrograms highlighted power modulations within the beta frequency range (13–30 Hz), characterized by substantial power reductions during both Delay and Response phases compared to the Fixation period. These patterns of beta-band modulation were most clearly visible in the normalized spectrograms.

### Event related desynchronization during the response phase in the beta-band

We observed a decrease in hippocampal beta-band power in the Response phase relative to the Fixation phase in at least one gray-matter channel in all participants using the cluster-based permutation test (*p* < 0.05) (Table 4). In total, 104 of the 149 (69.8%) electrode contacts in hippocampal gray matter showed a significant difference in beta-band power between these phases. Contiguous beta-band frequency bins with statistically significant power differences between the Response and Fixation phases after adjusting for the false discovery rate are presented in Table 4. This includes the average frequency range of statistically significant trial-averaged power modulation in addition to the number of electrode contacts in hippocampal gray matter that showed statistically significant power modulation for each patient. As mentioned, Fig. 3 shows the PSDs computed during each phase for participant 1, exhibiting a representative decrease in beta-band power in the Response phase.

**Table 4:** Statistical table of detailed comparison of beta-band modulation between response and fixation phases

Participant	Hand	Total	Loc	Hem	Contacts (%)	Avg freq	Max *p* value	Freq range
1	Right	7	Hippo	Left	100.0	22.3	0.046	13.0–30.0
	Right	8	Hippo	Right	100.0	22.4	0.046	13.0–30.0
2	Right	8	Hippo	Left	100.0	24.3	0.049	13.0–30.0
	Right	7	Hippo	Right	100.0	22.8	0.050	13.0–30.0
3	Right	4	Hippo	Left	100.0	23.9	0.047	13.0–30.0
	Right	4	Hippo	Right	100.0	23.9	0.042	13.31–30.0
4	Right	10	Hippo	Left	50.0	24.5	0.048	13.0–30.0
	Right	9	Hippo	Right	66.7	24.2	0.043	13.0–30.0
5	Right	9	Hippo	Left	22.2	22.6	0.044	21.04–24.44
6	Right	4	Hippo	Left	100.0	21.6	0.035	13.0–30.0
	Right	9	Hippo	Right	100.0	24.3	0.045	13.0–30.0
7	Right	11	Hippo	Left	36.4	25.5	0.049	20.11–30.0
	Right	5	Hippo	Right	40.0	26.1	0.050	20.42–30.0
8	Right	2	Hippo	Left	100.0	23.0	0.039	20.11–27.53
	Right	2	Hippo	Right	100.0	23.6	0.049	13.62–30.0
9	Right	6	Hippo	Left	16.7	27.1	0.049	21.96–30.0
	Right	3	Hippo	Right	33.3	26.4	0.050	22.27–30.0
10	Right	11	Hippo	Left	18.2	26.0	0.046	13.31–30.0
	Right	8	Hippo	Right	12.5	27.3	0.048	20.42–30.0
11	Right	13	Hippo	Left	92.3	23.0	0.048	13.0–30.0
	Right	9	Hippo	Right	77.8	22.0	0.046	20.11–24.14
Avg		7.1			69.8	24.1	0.046	18.26–29.33

A cluster-based permutation test was used to calculate the percentage of contacts in each location with significant modulation. There is an increase in percentage of contacts showing modulation compared to the Delay phase (average = 69.81%). The average median frequency range of contacts showing modulation is similar between two comparisons (both averaged around 24 Hz)

### Beta-band modulation between delay and response phase

We observed a decrease in hippocampal beta-band power in the Response phase relative to the Delay phase in at least one gray-matter channel in 3 out of 11 (27.3%) participants using the Wilcoxon signed-rank test (*p* < 0.05) (Table 5). In total, only 11 of the 149 (7.38%) electrode contacts in hippocampal gray matter showed a significant difference in beta-band power between these phases. Contiguous beta-band frequency bins with statistically significant power differences between the Response and Delay phases after adjusting for the false discovery rate are presented in Table 5. This includes the average frequency range of statistically significant trial-averaged power modulation in addition to the number of electrode contacts in hippocampal gray matter that showed statistically significant power modulation for each patient. As mentioned, Fig. 3 shows the PSDs computed during each phase for participant 1, exhibiting the trends in beta-band power for the Response and Delay phases.

**Table 5:** Statistical table of detailed comparison of beta-band modulation between Response and Delay phases

Participant	Hand	Total	Loc	Hem	Contacts (%)	Avg freq	Max *p* value	Freq range
1	Right	7	Hippo	Left	28.57	22.3	0.049	21.03–27.53
	Right	8	Hippo	Right	12.5	17.0	0.048	15.72–18.31
2	Right	8	Hippo	Left	25	22.7	0.048	21.03–24.43
	Right	7	Hippo	Right	28.57	21.3	0.05	20.41–22.27
3	Right	4	Hippo	Left	0			Nan–nan
	Right	4	Hippo	Right	0			Nan–nan
4	Right	10	Hippo	Left	0			Nan–nan
	Right	9	Hippo	Right	0			Nan–nan
5	Right	9	Hippo	Left	0			Nan–nan
6	Right	4	Hippo	Left	0			Nan–nan
	Right	9	Hippo	Right	11.11	29.38	0.049	28.76–30.0
7	Right	11	Hippo	Left	0			Nan–nan
	Right	5	Hippo	Right	20			Nan–nan
8	Right	2	Hippo	Left	0			Nan–nan
	Right	2	Hippo	Right	0			Nan–nan
9	Right	6	Hippo	Left	0			Nan–nan
	Right	3	Hippo	Right	33.33	24.74	0.048	23.81–25.67
10	Right	11	Hippo	Left	0			Nan–nan
	Right	8	Hippo	Right	0			Nan–nan
11	Right	13	Hippo	Left	0			Nan–nan
	Right	9	Hippo	Right	0			Nan–nan
Avg		7.1			7.58	22.91		21.80–24.70

A cluster-based permutation test was used to calculate the percentage of contacts in each location with significant modulation. 3 out of 11 participants (27.27%) displayed significant modulation in at least one contact in hippocampus, and 11 out of 149 contacts (7.58%) displayed significant modulation in beta-band

### Comparing hippocampal ipsilateral versus contralateral hemispheres

**Fig. 5:**
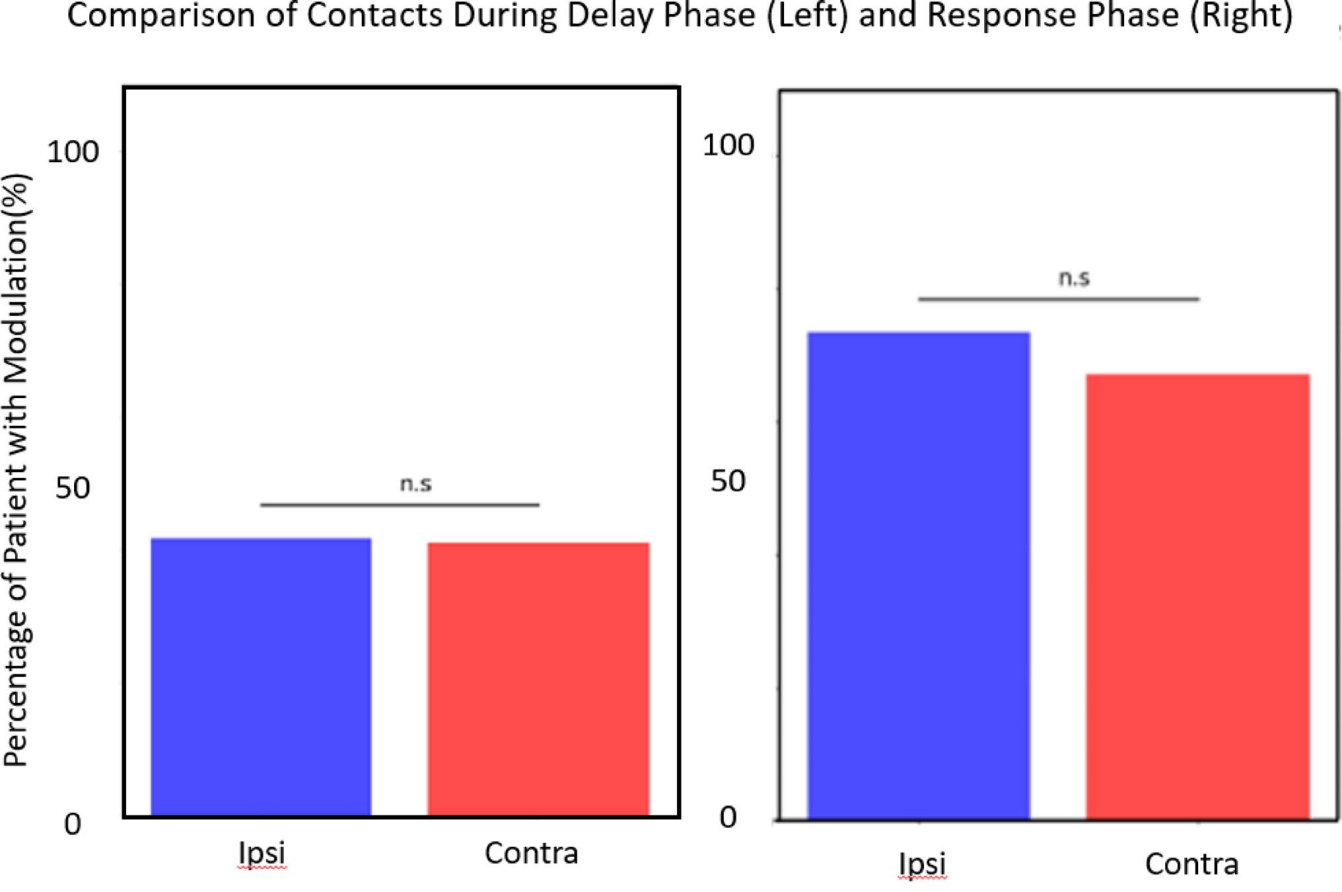
Comparison between the percentage of contacts with modulation in ipsilateral and contralateral in the hippocampus during the Delay phase (on the left) and during the Response phase (on the right). The histogram of the percentage of modulation of contacts in the ipsilateral and contralateral hemisphere (Yate’s z-test) showes no significant difference between ipsilateral and contralateral hippocampal modulation during the Delay phase (*p* > 0.05) or during Response phase (*p* > 0.05)

A supplementary comparison was made to examine the difference in beta-band modulation in the hippocampus based on laterality. Specifically, we compared ipsilateral and contralateral hemispheres of hippocampal gray matter. Using a cluster-based permutation test, we found that 31 of the 64 (49.0%) ipsilaterally implanted hippocampal contacts and 38 of the 85 (45.6%) contralateral hippocampal contacts showed significant beta-band modulation during the Delay phase (Table 6). There was no significant difference in the proportion of ipsilateral and contralateral hippocampal electrodes that showed modulation, with *p* = 0.12 (Fig. 5). During the Response phase, there was no significant difference in beta-band modulation between ipsilateral and contralateral contacts (*p* = 0.64). 47 of 64 (73.4%) ipsilateral contacts exhibit modulation, and 58 of 85 (68.2) contralateral contacts exhibit modulation (Fig. 5).

**Table 6:** Subanalysis of beta-band modulation in the hippocampus during the Delay phase in ipsilateral versus contralateral hemispheres

PAT	NO. IPSI/CONTRA ELE		IPSI/CONTRA %	
1	8	7	87.50	85.71
2	7	8	85.71	75.00
3	4	4	75.00	75.00
4	9	10	22.22	40.00
5	0	9	Nan	33.33
6	9	4	77.78	75.00
7	5	11	0.00	9.09
8	2	2	50.00	50.00
9	3	6	0.00	0.00
10	8	11	25.00	27.27
11	9	13	66.67	30.77
Avg	5.82	7.73	48.99	45.56

We observed no difference between ipsilateral and contralateral hemispheres

## Discussion

A growing body of research has suggested that the hippocampus may be involved in voluntary movement preparation and execution. In this study, we characterized the role of the beta-band in the hippocampus during a modified Delayed Reach center-out task reflective of voluntary movement preparation (Lara et al. 2018). In addition, we utilized a novel re-referencing method that takes the amplitude of each channel into account to avoid biasing the re-reference signal and remove artifacts.

### Hippocampus and the delay phase

We observed that 91% of participants and 46.8% of all hippocampal gray matter contacts exhibited significant decreases in beta-band power between the Fixation and Delay phases. Additionally, all participants and 69.8% of all contacts showed a significant decrease in beta-band power between the Fixation and Response phases, thus rejecting our null hypothesis. These results indicate that beta-band power in the hippocampus is significantly reduced from baseline during both the Delay and Response phases*.* Our finding of significant beta-band modulation between the Fixation and Response phases is consistent with prior studies from our lab that found beta-band modulation in the hippocampus during movement execution (del Campo-Vera et al. 2022; Gilbert et al. 2022). However, to the best of our knowledge, this is the first study characterizing beta-band power during movement delay in the hippocampus.

A significant component of the Delay phase is movement preparation, which involves the activation of neural circuits before the onset of movement. In the context of our Delayed Reach task, participants prepare their movement toward the target area during the Delay phase after learning the spatial location of the target circle. Several studies that have examined the role of the beta-band using similar movement tasks have found the onset of ERD during Delay phases in the motor cortex (Klostermann et al. 2007; Pfurtscheller et al. 1997; Tzagarakis et al. 2010) and subcortex (Alegre et al. 2005; Courtemanche et al. 2003; Kühn et al. 2004; Paradiso et al. 2004). Of note, Alegre et al. (2003) analyzed beta-band activity in the sensorimotor cortex during different kinds of stimuli presentation and found beta-band ERD only when a movement cue was periodic and predictable, not when it was aperiodic (Alegre et al. 2003). Since the only difference between both conditions was that the periodic stimuli were temporally predictable, the beta-band ERD they noted was most likely related to the ability to predict and prepare a movement (Alegre et al. 2003). Tzagarakis et al. (2010) expanded upon these observations by identifying that the reduction of beta-band power was scaled relative to the degree of directional uncertainty in an instructed-delay reaching task (Tzagarakis et al. 2010). In addition, Chung et al. (2017) found that greater beta-band desynchronization during motor control correlated with reduced movement error, and the authors suggested that the beta-band ERD responded to visual sensory feedback (Chung et al. 2017). Overall, these studies demonstrate that decreases in beta-band power, preceding voluntary movement execution, relate to movement preparation and can be modulated depending on the presence of stimuli affecting the certainty of that movement.

To the best of our knowledge, no prior studies have directly characterized the role of beta-band power in the hippocampus during movement preparation. Our results demonstrated the presence of beta-band ERD aligned to cue onset during the Delay phase of a Delayed Reach task, suggesting that the hippocampus may contribute to the movement preparatory activity consistent in cortical areas (Crammond and Kalaska 2000; Kalaska et al. 1997). Given the established hippocampal role in motor sequence learning (Albouy et al. 2008; Gheysen et al. 2010), Burman et al. (2019) sought to compare hippocampal connectivity during a sensorimotor task involving paced finger movements, one with a component of sequence learning and the other without (Burman 2019). They found hippocampal connectivity with sensorimotor areas during both tasks, indicating that the hippocampus is active during movement even in the absence of motor sequence learning (Burman 2019). Therefore, the effects of the beta-band may represent either direct modulation of movement preparatory activity by the hippocampus or signal propagation from connected sensorimotor areas.

The observed beta-band ERD during the Delay phase might be associated with the process of memory encoding, as participants are required to retain the spatial coordinates of targets within this interval. Several studies have indicated that beta-band may be decreased during memory encoding (Hanslmayr et al. 2012; Popescu et al. 2020; Rimmele et al. 2019; Subramaniam et al. 2019; Zammit and Muscat 2019). Other studies have reported relative increases in theta and gamma band power during memory encoding (Düzel et al. 2010). However, much of this previous work examined modulation in cortical regions rather than subcortical regions involved in memory such as the hippocampus. One study that attempted to characterize this modulation in subcortical regions was performed by Popescu et al. (2020). In their study, they utilized magnetoencephalography and demonstrated decreases in parahippocampal beta-band power during a *subsequent memory* paradigm in patients with post-traumatic stress disorder (Popescu et al. 2020). The duration of the task employed Popescu et al. (2020) was over several minutes (Popescu et al. 2020). Indeed, this aligns with the hippocampal role of being engaged in tasks necessitating delayed retrieval of working memory, particularly over extended delay durations (Friedman and Goldman-Rakic 1994; Zola-Morgan and Squire 1985). However, there is little evidence supporting its role in managing short-term delays (on the order of 2–4 s), with only reductions in the alpha-band frequency ranges being documented during increased working memory demands (Ames et al. 2019; Axmacher et al. 2010). Furthermore, it has been posited that beta-band ERD are reflective of semantic memory encoding processes while theta-band ERS are reflective of shallow encoding processes (Hanslmayr et al. 2009). Consequently, it is unlikely that the beta-band ERD we observed during the Delay phase (2–4 s) of our task in the hippocampus reflects spatial memory encoding. Nevertheless, because a designated control condition was not included in our task paradigm, future work is needed to characterize this on hippocampal beta-band oscillations.

In addition to motor preparation, an element of motor inhibition may also occur during the Delay phase of our task. Specifically, after identifying the location of the cue circle during the Cue phase, participants must hold movement toward the target area, due to condition constraints, until they receive the “Go” signal during the Response phase. Therefore, this situation may represent a “No-go” phase that imposes movement inhibition on participants. A previous study by our lab investigated the role of the beta-band in the hippocampus during a “No-go” task, and we found an increase in beta-band power during motor inhibition compared to motor execution during the “Go” condition (del Campo-Vera et al. 2022). In our Delayed Reach task paradigm, we observed significant beta-band ERD during the Delay phase in a vast majority of participants, suggesting that inhibitory processes are an unlikely explanation for the observed results. Rather, the beta-band ERD seen during the Delay phase is likely reflective of a movement preparatory signal of a pro-kinetic state, given that participants are aware of an upcoming planned volitional movement to the target upon cue presentation.

Attentional processing is another consideration that could impact hippocampal beta-band modulation during the Delay and Response phases. Indeed, beta-band oscillatory activity has been correlated with alertness (Gola et al. 2012) and with visual (Gola et al. 2013) attention. In particular, these scalp EEG studies observed that increases in beta-band power were associated with improved performance in behavioral attention tasks while decreases in beta-band power were correlated with worse performance (Gola et al. 2012, 2013). In our study, we observed decreases in beta-band power during the Delay and Response phases of the task when it was attentively and successfully performed by our participants. As such, it is likely that the beta-band ERD seen during these phases is more representative of an alternative process rather than that of decreased alertness or attention to the involved task (Gola et al. 2012, 2013). Nevertheless, much of the previous work regarding the beta-band in the hippocampus has been primarily focused on reward signaling in rats (Lansink et al. 2016) and novel object recognition in mice (França et al. 2014). Therefore, the relationship between hippocampal beta-band modulation and attentional processing is an area that requires further investigation.

When comparing beta-band modulation between the Delay and Response phases of our task, we observed relatively few significant differences. This suggests that the beta-band ERD in the hippocampus during the Delay phase may be consistent with the hypothesis that the beta-band acts as a state variable, promoting an existing motor set rather than directly responding to or initiating movement execution or inhibition. For example, Gilbertson et al. (2005) demonstrated that voluntary movements are slowed when they are initiated during periods of spontaneous beta-band power increases (Gilbertson et al. 2005). Similarly, Androulidakis et al. (2006) found that corrections in posture maintenance were more effective during periods of beta-band synchrony, suggesting that the beta-band may be involved in steady force output and is resistant to changes in movement velocity (Androulidakis et al. 2006). Additionally, Swann et al. (2009) showed that stopping movement during the “No-Go” phase of a Go/No-Go task is more successful during enhanced beta-band synchrony (Swann et al. 2009). Given these findings, Engel and Fries (2010) posited a unifying hypothesis about the overall role of the beta-band, suggesting that the beta-band serves to maintain the status quo (pro-kinetic/anti-kinetic) in the domain of motor control (Engel and Fries 2010). In our study, the beta-band modulation we witnessed in the hippocampus may represent its involvement in maintaining necessary motor control processes prior to motor execution, which is in line with its known roles in motor imagery and motor sequence learning (Albouy et al. 2008; Burman 2019; Fernández-Seara et al. 2009; Gheysen et al. 2010; Kerr et al. 2017; Shah et al. 2019). Furthermore, in our previous study, we examined beta-band ERD in the response phase of a direct reach center-out task (Del Campo-Vera et al. 2020). As the task did not involve a delay period, it may be that the beta-band ERD observed across both of our studies is reflective of preparatory activity that plays a straightforward, mechanistic role (Lara et al. 2018): initializing the networks involved in producing the downstream motor commands.

### Hippocampal sub-analysis

An intriguing avenue for further investigation pertains to the localization of movement-related neural signals. In prior research, we observed ERD in the beta frequency band in both contralateral and ipsilateral hemispheres during the Response phase of a direct reach task (Gilbert et al. 2022). Our analysis revealed a comparable pattern of beta-band ERD in both ipsilateral and contralateral hemispheres. While this does not definitively establish that the signal originates from both hemispheres, it does suggest that movement-related signals are not confined to a single hemisphere.

### Novel re-referencing method

Our study utilized a novel re-referencing approach by using RMS as a normalizing indicator to weight the contribution of each electrode contact to the common noise. Traditional methods for eliminating common noise in neural signals typically involve common average/median re-referencing (Gaona et al. 2011; Kubánek et al. 2009; Schalk et al. 2017), Laplacian re-referencing (McFarland et al. 1997; Nunez and Westdorp 1994; Shirhatti et al. 2016), bipolar re-referencing (Allen et al. 1992; Kobayashi et al. 2009; Vidal et al. 2012), and electrode shaft re-referencing. These techniques, however, weight each contact equally when calculating referencing signals. Therefore, using any of the above re-referencing methods will always result in a biased re-reference signal toward the contact with the largest RMS value. To our knowledge, this problem can also be addressed by scaling the signal during pre-processing to ensure no bias is introduced during re-referencing (Yong et al. 2008). However, this method will not be feasible during some cases where amplitude of the original signal is one feature to explore. In some cases, certain contacts that were higher in amplitude than others (usually contacts in gray matter), would have contaminated with the signals in lower amplitude contacts. Additional analysis in the supplementary material shows that compared to ESR and bipolar, this method results in the lowest off-diagonal correlation coefficient value for all contact pairs. Therefore, this novel methodology of scaling the weights based on the RMS of each contact’s neural activity avoided the problem of the software-computed reference biasing towards high-amplitude activity.

### Limitations and future directions

Our study has certain limitations to consider. Firstly, our data was collected from patients with chronic epilepsy, including three participants with seizure onset zones (SOZ) in the hippocampus. Although the hippocampus may retain functional capabilities despite involvement in seizure generation (Caciagli et al. 2019), the extent of network alterations in epileptic hippocampi remains not well understood (Klimes et al. 2016; Zhou et al. 2015). For our study, we opted to include all participants with electrode contacts in the hippocampus regardless of SOZ location to maximize statistical power and avoid potential selection bias. This approach is consistent with numerous studies from our laboratory (Campo-Vera et al. 2020; Chung et al. 2024; Gilbert et al. 2022) and others utilizing similar patient populations (Proix et al. 2022; Schwartz et al. 2023; Swann et al. 2009). Nevertheless, it remains unclear whether our findings would generalize to individuals without epilepsy. Therefore, additional investigations to determine whether this phenomenon is independent of epilepsy are warranted.

Secondly, given clinical constraints, electrodes were not implanted in our participants outside of structures hypothesized as being involved in seizure activity. As a result, we cannot determine how other brain regions without electrode implants interact with the hippocampus, nor how beta-band power globally evolves throughout movement planning and execution. Thirdly, common factors that could contribute to a global change in beta-band power regardless of movement include participant attention (Arif et al. 2023; Nguyen et al. 2017; Foong et al. 2015; Stancin et al. 2021), epileptic changes in the brain (Heers et al. 2014; Song et al. 2019), and distraction during the experimental task, which we were unable to control. Furthermore, our task paradigm did not include a condition that involved memory without motor preparation or a non-spatial task variant which would control for the potential confounds of memory encoding and spatial targeting respectively. Although the spatial targets were equally presented (each of the eight targets was presented randomly eight times), it remains possible that an aspect of spatial navigation encoding associated with hippocampal function could have impacted our results. Yet, prior work has shown that hippocampal delta, theta, and low-gamma oscillations are associated with successful spatial encoding, not beta oscillations (Park et al. 2014). Nevertheless, additional work is necessary to characterize these effects in more patients.

Despite providing forearm support with a pillow to create a near-resting position, our task required participants to maintain their hand at the center of the screen during key phases. This sustained posture could influence beta-band activity, as motor posture maintenance has been linked to increased beta oscillations (Jasper and Penfield, 1949). While we attempted to minimize this effect, some observed neural activity may reflect posture maintenance rather than purely cognitive motor preparation processes. Future studies should consider designs that further isolate these factors.

While our analysis focused primarily on beta-band oscillations (13–30 Hz), mu-rhythms (8–13 Hz), which share a similar frequency range with alpha oscillations but are specifically recorded over sensorimotor regions, demonstrate consistent desynchronization during both action observation and execution (Lapenta and Boggio 2014). These oscillations have been extensively linked to mirror neuron system activity, particularly during movement observation paradigms, complementing the role of beta oscillations in motor execution.

The topographical differences between mu and beta rhythms are notable, with beta showing a more diffuse focus around the vertex and mu-rhythms demonstrating specific sensorimotor localization (McFarland et al. 2000). Though our study targeted beta oscillations due to their established role in movement execution, future investigations could benefit from simultaneously examining both frequency bands to parse their distinct contributions to various aspects of movement processing. Such an approach might reveal how these oscillations interact across different phases of motor planning and execution, potentially offering a more integrated understanding of the neural mechanisms underlying movement control in the hippocampus and related structures.

Despite these limitations, we observed significant modulation in the beta-band in the hippocampus in the Delay phase of a Delayed Reach task. To our knowledge, this is the first study to investigate beta-band power in the hippocampus during a period involving movement preparation. Future lines of investigation could aim to further examine how beta-band activity evolves throughout movement fixation, delay, and execution in other structures of the brain that share connectivity with the hippocampus. Additionally, studies exploring how the beta-band activity scales with directionality and temporal uncertainty could provide further insights into the hippocampus’ role in motor processing.

## Supplementary Information

Below is the link to the electronic supplementary material.

Supplementary file 1 (PDF 265 kb)

## Data Availability

No datasets were generated or analysed during the current study.
