# Supplementary material for: Beta-band desynchronization in the human hippocampus during movement preparation in a delayed reach task
Source: Exp Brain Res. 2025 Jun 23;243(7):180. doi: 10.1007/s00221-025-07124-6 (PMC12185599; doi:10.1007/s00221-025-07124-6)
Supplement: Supplementary file 1 — (PDF 265 kb) [file 221_2025_7124_MOESM1_ESM.pdf]

Re-referencing Methods

To evaluate the performance of our weighted Electrode Shaft Re-referencing (weighted ESR) approach, we compared three re-referencing methods. The standard Electrode Shaft Re-referencing (ESR) calculates the common average reference as the mean of all contacts within the same electrode shaft. Bipolar referencing computes signal differences between adjacent contacts on the same shaft. Weighted ESR approach applies RMS-based weighting factors to each contact when calculating the common average reference, giving less influence on contacts with higher amplitude. We visualized the performance of these methods using Pearson correlation coefficient matrices between all electrode contacts (Figure S1). The standard ESR showed higher off-diagonal correlation (0.058). Bipolar referencing displayed the lowest global correlation (0.05) but introduced strong correlations between adjacent contacts. The weighted ESR achieved moderate off-diagonal correlation (0.49) while preserving physiologically meaningful signal correlations.

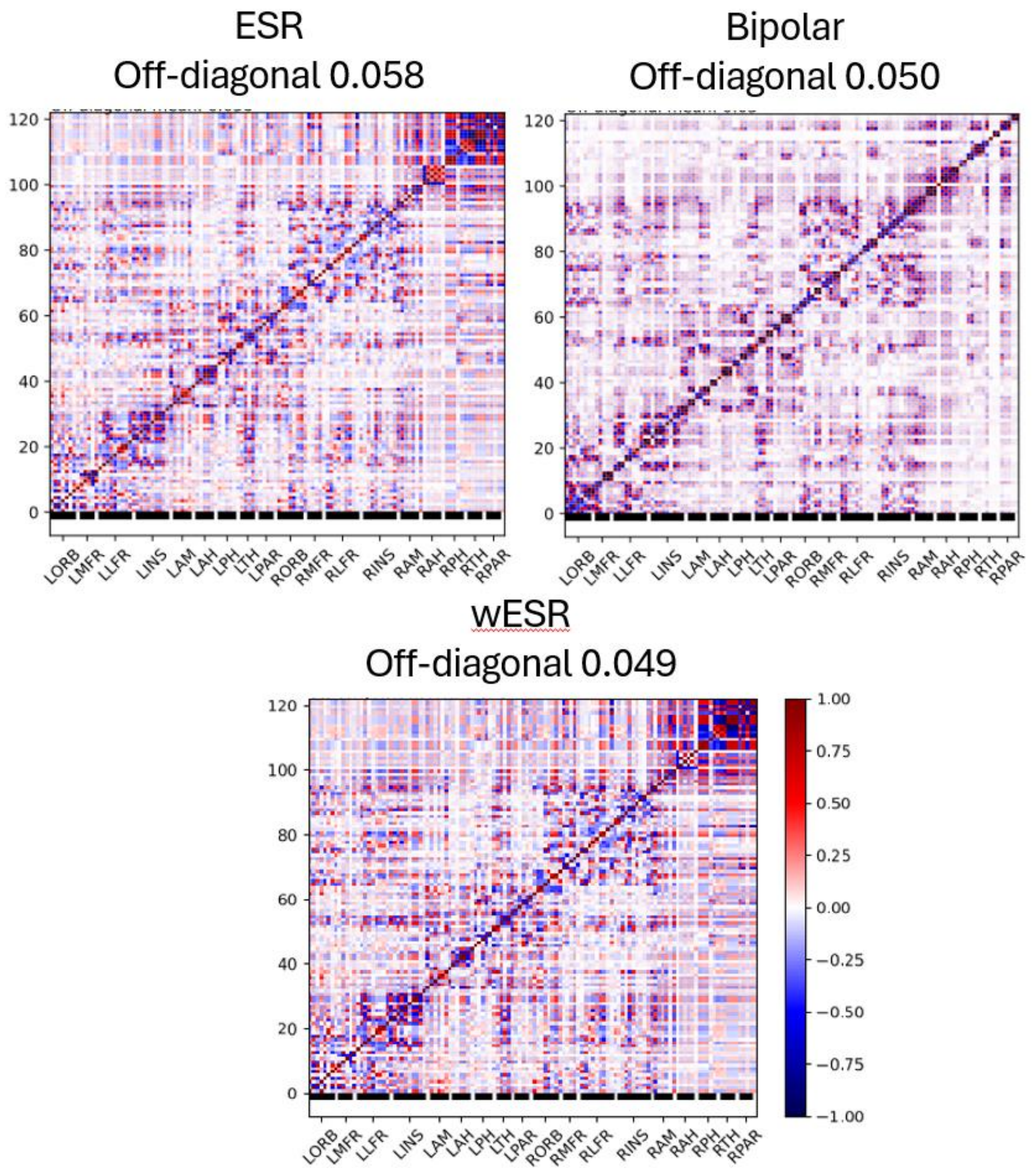

Figure S1: Comparison of electrode correlation matrices for different re-referencing methods. Pearson correlation coefficient matrices showing the similarity between electrode contacts for three different re-referencing techniques: standard Electrode Shaft Re-referencing (ESR, left), bipolar referencing (right), and weighted ESR (bottom). Color scale represents correlation coefficient values ranging from -1.0 (dark blue) to 1.0 (dark red). The off-diagonal mean correlation value is displayed in the title of each panel. Standard ESR shows a higher off-diagonal correlation (0.058. Although bipolar referencing shows reduced global correlation (0.05) but the high correlation coefficient between adjacent contacts. The weighted ESR method achieves moderate off-diagonal correlation (0.049) while preserving physiologically meaningful signal correlations. Bottom x-axis shows different electrode locations.
